# Indigenous Foods to Address Malnutrition: An Inquiry into the Diets and Nutritional Status of Women in the Indigenous Community of Munda Tribes of Jharkhand, India

**DOI:** 10.1093/cdn/nzac102

**Published:** 2022-09-13

**Authors:** Suparna Ghosh-Jerath, Ridhima Kapoor, Ashish Bandhu, Archna Singh, Shauna Downs, Jessica Fanzo

**Affiliations:** Indian Institute of Public Health-Delhi, Public Health Foundation of India, Gurgaon, India; Indian Institute of Public Health-Delhi, Public Health Foundation of India, Gurgaon, India; School of Institute of Health Management Research, IIHMR University, Jaipur, India; Department of Biochemistry, All India Institute of Medical Sciences (AIIMS), New Delhi, India; Department of Urban-Global Public Health, Rutgers School of Public Health, New Brunswick, NJ, USA; Berman Institute of Bioethics, Nitze School of Advanced International Studies (SAIS) and Bloomberg School of Public Health, Johns Hopkins University, Washington, DC, USA

**Keywords:** nutrient intake, nutritional status, indigenous food consumption, dietary diversity, Munda women, indigenous communities, Indian tribal women

## Abstract

**Background:**

Indigenous people globally experience poor nutrition outcomes, with women facing the greater burden. Munda, a predominant tribe in Jharkhand, India, live in a biodiverse food environment but yet have high levels of malnutrition.

**Objectives:**

To assess diets and the nutritional status of Munda tribal women and explore associations with their Indigenous food consumption, dietary diversity, and socioeconomic and demographic profiles.

**Methods:**

A cross-sectional study with a longitudinal component to capture seasonal dietary intake was conducted in 11 villages of the Khunti district, Jharkhand. Household surveys and FFQs, supplemented with 2-d 24-h dietary recall and anthropometric assessments on 1 randomly selected woman per household were conducted.

**Results:**

Limited access to diverse foods from a natural food environment (Food Accessed Diversity Index score of 0.3 ± 0.3) was observed. More than 90% women in both seasons had usual nutrient intakes below the estimated average requirements for all nutrients except protein and vitamin C; 35.5% of women were underweight. The mean Minimum Dietary Diversity Score among women (MDDS) was low [2.6 ± 0.6 in wet monsoon; 3 ± 0.7 in winters (acceptable ≥5)]. Higher MDDS contributed to higher usual nutrient intakes (*P* <0.001). Indigenous food intakes in both seasons (wet monsoon and winter) were low, e.g. Indigenous green leafy vegetables [10.5 and 27.8% of the recommended dietary intake (RDI), respectively], other vegetables (5.2% and 7.8% of RDI, respectively), and fruits (5.8 and 22.8% of RDI, respectively). Despite low intakes, the Indigenous food consumption score was positively associated with usual intake of vitamin A, riboflavin, vitamin C, pyridoxine, and calcium (*P* < 0.05) in the wet monsoon and thiamine, riboflavin, and zinc (*P* < 0.001) in winters. After adjusting for covariates, Indigenous food consumption was associated with a higher usual intake of vitamin A (*P*  < 0.001) in the wet monsoon season.

**Conclusion:**

Contextual food-based interventions promoting Indigenous foods and increasing dietary diversity have the potential to address malnutrition in Munda women.

## Teaser

Dietary diversity and Indigenous food consumption for addressing malnutrition in women belonging to the Indigenous Munda community of India.

## Introduction

There are an estimated 476 million Indigenous Peoples worldwide, who possess their own social, economic, cultural, and political characteristics and reside within geographically distinct traditional habitats (1, 2). These Indigenous populations manage over 25% of the world's land surfaces and support nearly 80% of the global biodiversity (3). In India, Indigenous populations constitute 8.6% of the total national population, which are divided across 705 individual Indigenous communities and are recognized as “Scheduled Tribes” by the Indian Constitution (4). Several of these Scheduled Tribes live in close vicinity of forests and have been playing their part in protecting and conserving biodiversity for hundreds of years (5). Despite their rich traditional ecological knowledge (TEK), access to biodiverse resources, and continued support from tribal welfare schemes, the tribal communities in India have a compromised nutritional status and a dismal quality of life owing to factors such as geographical isolation, financial insecurity, poor literacy, suboptimal living conditions, and inadequate access to health and social services (6–13).

The impact of these factors is reflected in the poor health and nutritional status of Indian tribal communities, particularly among young children and women of reproductive age (14–16). According to the National Family Health Survey (2015–2016), the prevalence of chronic energy deficiency (CED) and anemia among women aged 15–49 y is higher in the tribal population compared with the corresponding national figures for the general population (31.7% versus 23% for CED and 59.9% versus 53.1% for anemia) (14). Similarly, at the state level, studies conducted in parts of Jharkhand, West Bengal, and Chhattisgarh have reported a high prevalence of CED (ranging from 27% to 50%) among tribal women (10–12, 17–19). The surveys conducted by the National Nutrition Monitoring Bureau during 2008–2009 in the states of Odisha, West Bengal, Tamil Nadu, and Madhya Pradesh have also documented poor diet quality, nutrient intakes, and several micronutrient deficiencies among tribal women (16).

Jharkhand, a central eastern state of India, has a high proportion of tribal communities (20). It is home to 32 tribal communities which constitute 26.2% of the total state population. Mundas are one of the predominant tribes in Jharkhand that account for 14.8% of the total state tribal population, and mainly inhabit the Chotanagpur region (21, 22). The community is mainly dependent upon subsistence agriculture, and engages in foraging, hunting, and livestock breeding to supplement their economy (23). They are surrounded by rich diverse ecosystems, possess TEK and are known to consume wild Indigenous foods comprised of culturally valued plant, animal, and fungi species (23, 24). However, despite being surrounded by rich agroforestry, studies have reported high levels of malnutrition among Munda women and children due to their extreme poverty and poor knowledge in regard to accessing and consuming nutritious foods from those sources (25, 26). Hence, there is an urgent need to preserve TEK regarding Indigenous foods of the Munda tribe which must be combined with data on their nutrient content to build food-based strategies for addressing widespread malnutrition in the community (27, 28).

For generations, Indigenous Peoples’ traditional food systems have maintained human health and natural environments (29). However, these biodiverse resources may not have received enough attention during the state's land-use planning and implementation, economic development, and biodiversity conservation and as a result, this TEK is rapidly diminishing due to urbanization, industrialization, and lifestyle changes (22). Dietary diversification [a strategy involving the incorporation of diverse food groups in a diet to improve micronutrient intake (28, 30)], along with a special focus on homestead food production and Indigenous food (IF) consumption, could improve health and nutritional well-being and may also offer social, economic, and environmental benefits for Indigenous communities (31–34). In this article, we aimed to understand the diets and nutritional status of Munda tribal women, with a special focus on their IF consumption and dietary diversity. There are multiple interconnected factors at the individual, family, community, and environmental level that may influence the consumption of diverse IFs (35) and their contribution to nutritional status. The association of the various sociodemographic, economic, and other factors related to the food environment of the Munda tribal community on dietary intake and nutritional status of the women were also explored in this study.

## Methods

The work shown in this article is part of a larger project with an overarching objective of exploring the IF systems of tribal communities of Jharkhand and understanding their impact on the nutritional status of tribal women and children. A detailed study protocol of the project is reported elsewhere (36). In the present article, we report the dietary intake and nutritional status of Munda tribal women and explore the associations between IF consumption, dietary diversity, and nutrient intake of women while considering other sociodemographic and environmental factors.

### Study area

**FIGURE 1:**
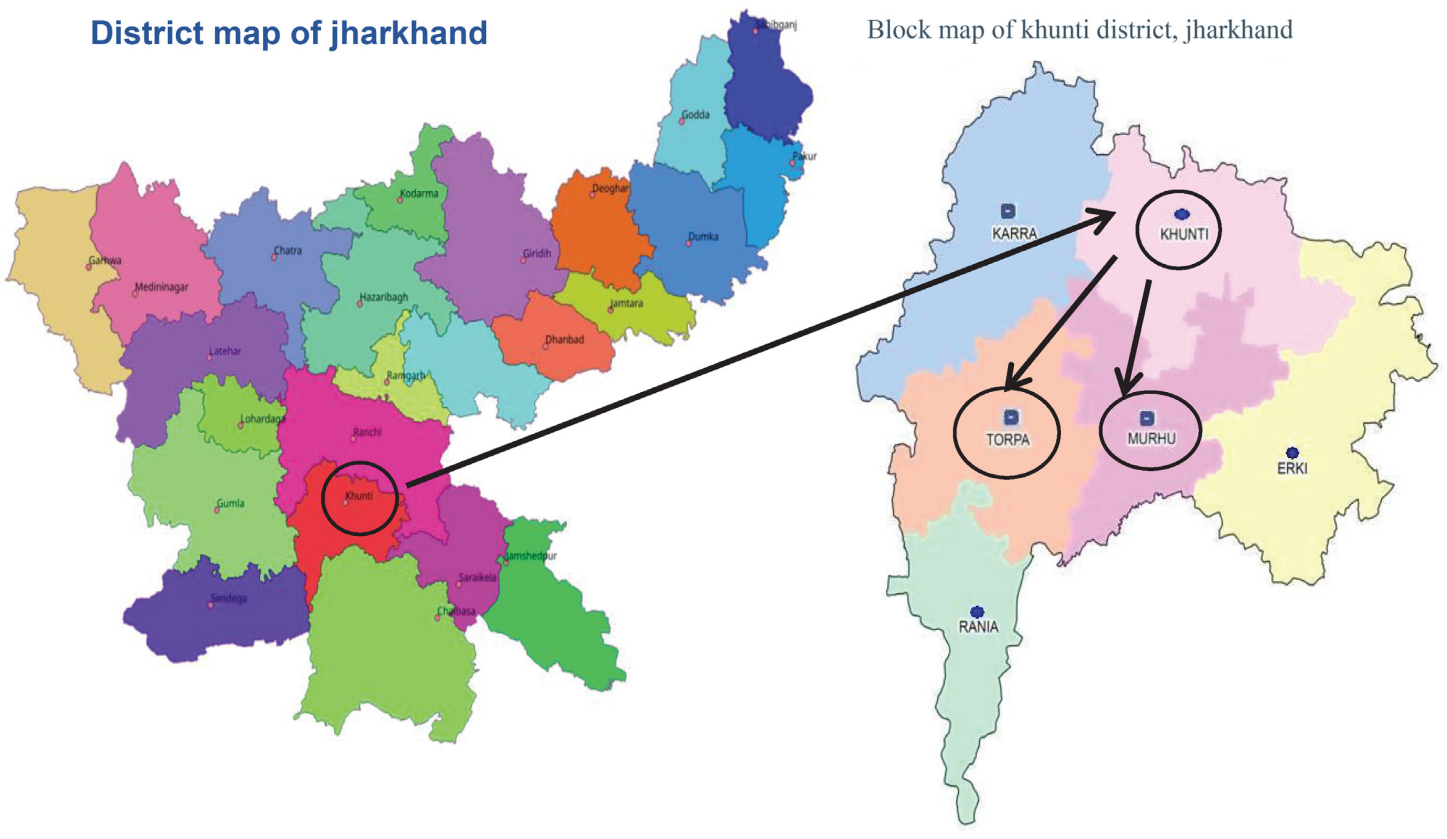
Selection of Murhu and Topra blocks from the Khunti district of Jharkhand, India.

This study was conducted in the Khunti district in Jharkhand, India, which is mainly inhabited by the Munda community [61% of the total district population (37)]. Two geographically distinct blocks of the Khunti district namely, Murhu and Torpa, with predominantly higher tribal populations were purposefully selected for the study (**Figure 1**).

### Study design and period

This was a cross-sectional study with a longitudinal component to capture seasonality in the dietary intake patterns of Munda tribal women. The dietary data were collected in 2 seasons—the wet monsoon (August 2019) and winter season (January 2020). First, a detailed household (HH) survey was conducted with 1 eligible woman of reproductive age (18–49 y) per HH during the monsoon season. This was also supplemented with a 24-h dietary recall (24-h DR) on 2 non-consecutive days and anthropometric assessments. A subset of the study sample (more than one-third of the same women) were subsequently followed up in the winter season with 24-h DR to capture seasonal variations in food and nutrient intakes.

### Sampling framework and study population

**FIGURE 2:**
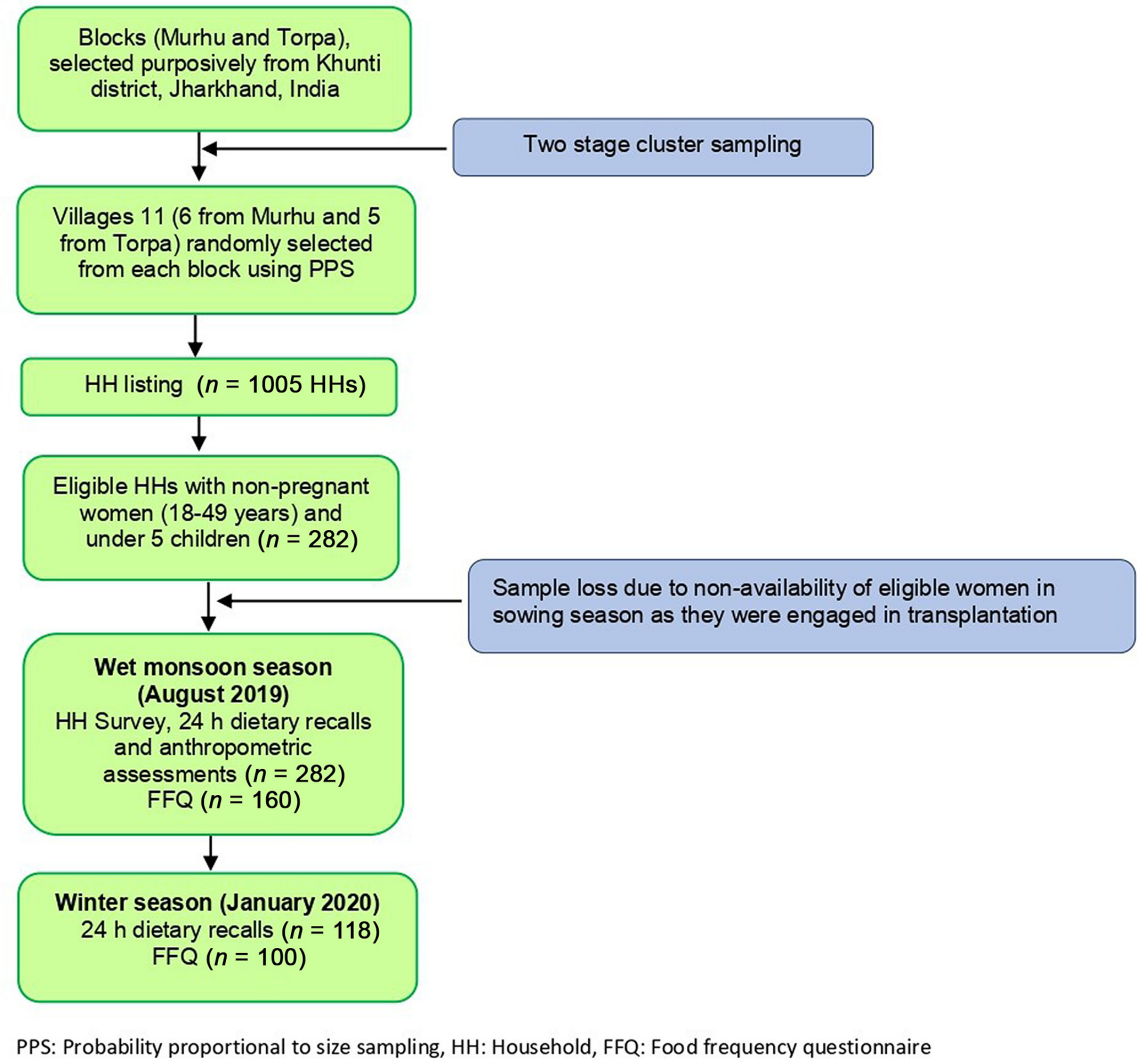
Flow diagram depicting data collection at various stages of this study.

A 2-stage cluster sampling design was followed (**Figure 2**). In the first stage, 2 blocks in Khunti district (Murhu and Torpa) were purposively chosen based on their accessibility and geographical distribution. Using a tribal village list from Census 2011 (20), 11 villages (6 from Murhu and 5 from Torpa) were selected using probability proportional to size (PPS) sampling. In the second stage, all 11 selected villages were visited, and a house-listing exercise was conducted to construct the sampling frame of all eligible HHs in the Munda community. Eligibility was based on the presence of 1 non-pregnant woman in the reproductive age group (18–49 y) and 1 child (6–54 mo) in the HH. In the case of >1 eligible women in a HH during the house listing, 1 woman was randomly selected for the interview using the Kish table (38).

### Sample size calculation

The sample size was calculated based on the difference in mean dietary iron intake of 3.8 mg/d (35% increase) with an SD of 7 mg/d between consumers and non-consumers of IFs, reported in a previous study among Santhal tribal women in Jharkhand (39). In order to detect the suggested minimum difference in iron intake between consumers and non-consumers of IFs, a minimum of 134 women in each group were required to be sampled with 80% power and a 5% level of significance. A design effect of 2.0 was also used to account for the loss in precision due to cluster sampling. Based on the above calculation, a total sample size of 268 women was derived for the present study. To achieve the requisite sample size, HH listing was done in 1005 HHs, out of which 309 HHs were found to be eligible for our survey. Among these eligible HHs, the data collection (HH, dietary assessment, and anthropometry surveys) was completed in 282 HHs in the monsoon season (which coincided with the agricultural activities of the community) and seasonal dietary surveys (24-h DR) were completed in more than one-third (41.8%) of the same women (*n* = 118) in the winter season (Figure 2).

### Data collection tools

#### Household survey

A pretested computer-assisted personal interviewing (CAPI) HH survey questionnaire was administered in all selected HHs by trained local field investigators to elicit information on the socioeconomic and demographic profile of HHs and their access to different food sources such as the natural food environment (cultivated food sources like farms and kitchen gardens, and wild food sources like local forests, water bodies, roadsides, and wastelands/unusable lands) and the built food environment (local markets, village corner shops, and the government's food supplementation and food security programs). The HH questionnaire was developed and collected on an electronic data capture platform using a Samsung tab (Model SM-T385) and the software CS pro (Version 7.2), that had provision for extensive built-in checks (context, range, and logic checks) to maintain data quality.

#### Food Frequency questionnaire

The frequency of consumption of different food items under various food groups was assessed using an FFQ. The food items included in the FFQ were identified during focus group discussions with the study community (23) (conducted in June 2019) and included both commonly consumed IFs [detailed list provided in (23)] as well as non-IFs. The FFQ was administered at HH level by trained nutritionists who inquired about the frequency of food consumption over the past 1 mo without specification of portion sizes. Nine predefined frequency categories ranging from “never” to “2 or more times per day” were used. Based on the seasonal availability of food items, during the monsoon season, a 318-item FFQ was administered in 160 HHs and during the winter season, a 257-item FFQ was administered in 100 HHs.

#### 24-h Dietary recall

A non-consecutive, 2 days multipass 24-h DR was conducted by trained nutritionists assisted by field investigators in monsoon and winter seasons, to capture realistic dietary intakes of 1 selected woman per HH. For the DR, the selected woman was asked to recall and report details of her food intake for each meal eaten during the past 24 h prior to each day of survey. A culturally appropriate flipbook was used with pictorial representation of portion sizes of food items (fruits, vegetables, and packaged and miscellaneous foods) which were locally available during the respective season. Use of images of local foods during the 24-h DR not only helped objectively in the assessment of dietary intake by reducing recall bias by the respondents, but also provided valid estimates of dietary intakes (40–42). To minimize misreporting of quantities, we used standard measuring cups and spoons for estimating the HH measures of the ingredients for recipes and consumed food items. A detailed description of the recipes of home-cooked meals as well as names for each ingredient consumed along with amounts, method of preparation, brand name etc. were gathered.

#### Anthropometry

Anthropometric measurements were taken to assess the nutritional status of the women. These measurements were carried out using standard protocols (43) and equipment [weighing scale (Seca Model 813) and stadiometer (Seca Model 213)] to measure BMI on the same women from whom dietary recalls were taken.

### Measurement of variables

The outcome variables for this study included usual intakes of macro- and micronutrients and the anthropometric status of women. The exposure variables included IF consumption at the individual level, Minimum Diet Diversity Score for each woman (MDDS), the HH Food Accessed Diversity Index, the HH Wealth Score Index, and educational profile of Munda women. These variables are briefly explained below:

#### Exposure variables

1. **IF consumption at the individual level:** The list of food items reported in the 24-h DR were used to calculate the IF consumption score for the selected participating women. If a woman consumed any IF over the recall period of 2 d, she was considered as an IF consumer and given a score based on the number of IFs consumed in a day (irrespective of the food amount consumed). Scoring for IF consumption was based on food groups, i.e. if a woman consumed 2 IFs from the same food group, she was given a score of 1, whereas if a woman consumed 2 IFs from different food groups, she was given a score of 2. The identification of IFs among the Munda community was based on a free food list obtained through focus group discussion conducted in previous surveys, which was followed by categorizing the foods as IFs, non-indigenous, and hybrid varieties based on the discussions with the community and literature search (23). The detailed list of IFs known and consumed by the Munda community are reported elsewhere (23).
2. **MDDS:** The 24-h DR data were further utilized to calculate MDDS—an index which is specifically designed for use with women of reproductive age and serves as a proxy for the probability of micronutrient adequacy (44). MDDS was computed by summing the total number of food groups (for food items with amounts ≥15 g) consumed by the woman as reported in the 24-h DR. The final MDDS was calculated by averaging the total food groups consumed by the woman over the previous 2 d. Women who consumed ≥5 of the 10 food groups were considered to be meeting the recommendations for minimum dietary diversity.
3. **Food Accessed Diversity Index:** For assessing agroforestry diversity at the HH level, the HH survey data on total number of foods accessed from cultivated and wild food sources were used to obtain the Food Accessed Diversity Index (FADI) (11, 45). This index was calculated by dividing the total number of foods grown, gathered, or accessed (excluding market foods) as well as animals raised in a particular HH by the corresponding maximum possible number of foods grown, gathered, accessed, and raised in a particular village (*N*).

The FADI was expressed as:

(1) $$\begin{eqnarray*}
{\rm{FADI = }}{\left( {\frac{{\rm{\mathit{ n}}}}{{\rm{\mathit{ N}}}}} \right)}^{\rm{2}}
\end{eqnarray*}$$

4. **HH Wealth Score Index:** HH characteristics such as type of house, number of rooms, presence of a separate kitchen and toilet, source of electricity and drinking water, possession of another house, kitchenware, and monthly expense on food items were elicited and used to generate the HH Wealth Score Index. This variable was computed using principal component analysis (PCA), an adaptive statistical technique for reducing the dimensionality of large data sets.
5. **Educational profile of women**: The category for the women's educational profile was recategorized into: *1*) primary level and below, *2*) above primary but below secondary level, and *3*) secondary level and above.

#### Outcome variables

The food intake data from 24-h DR on 2 non-consecutive days were converted to macro- and micronutrient intakes using a validated software “DietCal” (Version 10.0; Profound Tech Solution), which is based on nutritive values of foods from the Indian Food Composition Table (IFCT), 2017 (46). The usual nutrient intakes obtained after due statistical transformations (explained in the next section) were considered as an outcome variable and used for further analysis.

The other outcome variable was obtained from the anthropometric measurements, that were used to compute BMI values [weight (in kg) divided by height (in m^2^)]. These values were used to classify women as being underweight and having CED or not, using standard BMI cut-offs (47).

### Statistical analysis

Since most of the nutrient intakes were non-normally distributed, the box-cox transformation method was used (48). The transformed 2-d 24-DR data on macronutrient and micronutrient intakes were then used in a linear mixed effect regression model. The values predicted using the linear mixed effect regression model were back-transformed to compute the “usual nutrient intake” (49). The usual nutrient intakes obtained were also observed to be skewed and hence were again transformed using the box-cox procedure. The estimated average requirement (EAR) cut-point method (50) was used to estimate the prevalence of nutrient inadequacy in the study population, by assessing the proportion of women whose usual nutrient intake fell below the EAR for moderately active Indian women (51). In the case of iron intake, the full probability approach was used for estimating nutrient inadequacy (50). We used univariate analysis to describe participants’ sociodemographic and economic characteristics. The HH Wealth Index Score was obtained by performing PCA using the statistical software Stata version 15.1 (52). The HHs were then classified into 5 groups based on the quintiles of the HH Wealth Index Score. The continuous variables were reported as mean ± SD, and categorical variables were summarized using frequencies and percentages. We employed bivariate analysis using the Karl Pearson correlation coefficient, *t*-test, and 1-factor ANOVA for association between exposure variables and outcome variables (53). Further, the linear mixed effect regression model was developed to estimate the effect of exposure variables. Owing to the nested structure of the data, the linear mixed effect regression technique with villages as the random effect was used to investigate the linear relation of usual intake of each nutrient with IF consumption and MDDS adjusting for other factors such as HH Wealth Score Index and education profile of women as fixed effects. Multicollinearity was assessed using the variance inflation factor. To assess the seasonal effect, all the covariates (as described above) were used in the linear mixed effect regression but season was additionally used as a fixed effect. A 2-tailed α of 0.05 was considered statistically significant. All data were analyzed using Stata version 15.1 (52).

### Ethical considerations

This study was conducted according to the guidelines laid down in the Declaration of Helsinki (54) and all procedures involving human subjects were approved by the Institutional Ethics Committee at the Indian Institute of Public Health–Delhi, Public Foundation of India and All India Institute of Medical Sciences, New Delhi. Written administrative approvals from authorities at the district level and cluster level consent from village leaders were obtained prior to data collection. Before survey commencement, all study participants were informed about the study objectives and were assured that the data collected from them would be kept safe and secure by the research team, and any identifiable data will be anonymized at the stage of analysis. The participants were also informed that all study findings would be shared with the community and would be used in published research and reports. Based on this information, a written consent was taken from literate participants and a verbal witnessed consent was taken from illiterate participants in the presence of a third party. Participation in the study was voluntary and incentives in the form of a small gift were given to the selected women of the HHs.

## Results

### Household profile of the Munda community

The HH survey revealed that Munda tribes had suboptimal living conditions in terms of housing, use of cooking fuel, and place of defecation (**Table 1**). The head of the HHs were usually males in majority of the HHs (78%), with a mean family size of 5.9 ± 2.1. Settled agriculture was reported as the primary occupation in the HHs (80%) and only a couple of HHs were primarily engaged in waged labor (8%). The majority of the HHs followed *Sarna* religion (worship of nature), followed by Christianity (46%) and Hinduism (3%). Literacy rates were low, with only one-third of the heads of the HH and selected women participants having a primary level of education.

**TABLE 1:** Social-demographic and economic profile of the households of Munda tribal community, Jharkhand, India (*n* = 282)

Characteristics	*n* (%)
**House type**	
*Kaccha* (mud/thatched roofs and walls)	245 (86.9)
Semi-*Pakka* (semi-cemented roofs and walls)	27 (9.6)
*Pakka* (cemented roofs and walls)	10 (3.5)
**Source of drinking water**	
Well	206 (72.9)
Tube well/hand-pump	54 (19.2)
Pond	7 (2.5)
Piped water	7 (2.5)
Others	8 (2.9)
**Source of cooking fuel**	
Firewood/chips/grass/stems/straw/shrub/agriculture waste	275 (97.5)
Liquid petroleum gas (LPG)	5 (1.8)
Gobar gas/biogas	2 (0.7)
**Defecation**	
Open field/forest	179 (63.4)
Personal toilet	102 (36.2)
Public toilet	1 (0.4)
**Source of light**	
Electricity	174 (61.7)
Kerosene	96 (34)
Solar panels	12 (4.3)
**TPDS ration card** ^1^	
Red (BPL)	120 (42.5)
Yellow (AAY)	91 (32.3)
Green (APL)	1 (0.4)
Don't have	70 (24.8)
**Religion**	
*Sarna* (worship of nature)	142 (50.4)
Christian	130 (46.1)
Hindu	10 (3.5)
**Family type**	
Joint	138 (48.9)
Nuclear	116 (41.1)
Extended	28 (10)
**Gender of household head**	
Male	220 (78)
Female	62 (22)
**Literacy level of household head**	
No formal education	101 (35.9)
Less than primary (until 4th standard)	19 (6.7)
Primary but less than secondary (until 9th standard)	111 (39.3)
Secondary (10th standard) & above	51 (18.1)
**Occupation of household head**	
Settled agriculture	227 (80.5)
Daily wager (agriculture & non-agriculture)	23 (8.2)
Service (government and private)	13 (4.6)
Housewife	13 (4.6)
Unemployed	4 (1.4)
Craftsmen/artisans/self-employed	2 (0.7)
**Educational level of selected women**	
No formal education	115 (40.8)
Less than primary (until 4th standard)	21 (7.5)
Primary but less than secondary (until 9th standard)	95 (33.7)
Secondary (10th standard) & above	51 (18)
**Age of selected women in years** mean ± SD	28.6 ± 6.6
**Number of family members** mean ± SD	5.9 ± 2.1
**Food Accessed Diversity Score** mean ± SD	0.3 ± 0.3

1 Targeted Public Distribution System (TPDS) is a national food security scheme in India, under which, major food commodities like rice, wheat, sugar, salt, and/or kerosene oil are distributed to poor households at subsidized rates. Possession of a PDS ration card (an official document entitling the holder to receive food ration) under various categories of poverty, that is, APL (Above Poverty Line), BPL (Below Poverty Line), and AAY (Antyodaya Ann Yojana), a category based on degrees of poverty, entitles the household to access the food product at highly subsidized rates.

### Availability and access to different food sources

#### Natural food environment

Most HHs owned agricultural lands (94%) and reported practicing settled agriculture at 3 levels of farmlands, namely, *Loyong* (low level lands with the highest water requirement for crops) (94%), *Badi* (middle level lands with a relatively low water requirement for crops) (74%), and Goda (dry stony plain lands with the least water requirement) (79%). The HHs typically grew crops such as rice (both Indigenous and hybrid varieties), pulses (horse gram, red gram, and black gram), and certain vegetables and tubers (tomato, cucumber, brinjal, potato) which were mainly used for home consumption. An additional 74% of HHs further reported accessing wild open spaces like roadsides and wastelands/unusable lands for collecting wild Indigenous green leafy vegetables (GLVs) (green Amaranth, red Amaranth, *Ponnaganni*, creeping marsh weed, Pot Cassia, *Punarnava*,*Hurhura*, and *Bathua* leaves) that grow as weeds. Munda HHs also reported growing GLVs and other vegetables in kitchen gardens (55%) and raising livestock (89%) such as goats, poultry, and pigs to produce home-grown foods for HH consumption. The Munda HHs also gathered food from wild sources like local forests (80%) and water bodies (ponds/rivers) (73%) to collect wild fruits, vegetables, roots and tubers, mushrooms, and Indigenous fishes, snails, prawns, crabs, and turtles for HH consumption. The hunting (albeit once a year) of wild animals and birds such as porcupine, spotted dove, wild birds like *Duhur*, *Askal*, and *Sursuri* was also reported by 60% of HHs. Despite the availability of diverse natural food sources, we observed poor diversity in production and access of individual food items. The mean FADI score of HHs was found to be quite low (0.3 ± 0.3, range: minimum—0, maximum—1), and only 4% HHs (*n* = 11/282 HHs) had a FADI score of 1, whereas the majority of HHs (75%) had a FADI score <0.44.

#### Built food environment

The community also reported food access from the built food environment, which included informal markets [local weekly markets (*Haats*) and village corner shops] and formal markets including government food security and supplementary feeding programs. All the HHs reported accessing *Haats* to purchase foods like rice, pulses, vegetables, roots and tubers (mainly potatoes, onion, and garlic), cooking oil, and spices. Apart from this, about three quarter of the HHs accessed formal markets like fair price shops [under the Targeted Public Distribution System (TPDS), a government food security program that distributes subsidized items (such as rice, wheat, sugar, salt, kerosene oil, etc.) to poor HHs (55)]. In addition, 84% of HHs accessed supplementary feeding programs (under Integrated Child Development Services) to avail hot cooked meals and take-home rations for preschool children and about 64% of HHs further reported access to the Mid-Day Meal program [a school meal program in India that provides free mid-day meal on working days for children studying in primary and upper primary classes in government schools (56)].

### Food consumption pattern of adults in Munda Households

Analysis of the FFQ data showed the HH-level consumption of food items accessed from natural and built food environments (**Table 2**). It was found that the majority of the Munda HHs in both monsoon and winter seasons consumed rice as a staple twice daily, however, the consumption of Indigenous rice varieties was relatively low (24% in monsoon and 61% in winters). Vegetables, roots, and tubers were also consumed habitually by the community; however, only one-quarter of the HHs in both seasons reported the weekly consumption of Indigenous vegetables, whereas few HHs (31% HHs in monsoon and 20% in winters) reported the weekly or a fortnightly consumption of wild tubers. Most HHs reported the consumption of pulses and GLVs once or twice a week in both seasons, among which mainly Indigenous varieties were consumed (56% for pulses and GLVs in monsoon, and 38–41% for pulses and GLVs in winters). The consumption of Indigenous fruits was found to be infrequent; more than half the HHs in both seasons (63% in monsoon and 61% in winters) did not consume any wild Indigenous fruit variety in the past month. Further, the weekly consumption of flesh foods (meat, poultry, and fish) in both seasons was reported, although the consumption of wild game was observed to be rare, with less than one-third of the HHs (31% in monsoon and 22% in winters) reporting once a month consumption. The majority of the HHs in both seasons did not consume any milk or milk products. Packaged and freshly prepared foods from local markets (like biscuits, chips, namkeen, sweets, pakodas, chowmein etc.) were reportedly consumed once or twice a week, although higher consumption (61% compared with 34%) was observed during the monsoon season.

**TABLE 2:** Frequency of food group consumption at household level during wet monsoon (*n* = 160 HHs) and winter season (*n* = 100 HHs) in Munda tribal community, Jharkhand, India

**Frequency of consumption** ^1^ **, *n* (%)**												
*** Food group***	**Wet monsoon season (*n* = 160 HHs)**						**Winter season (*n* = 100 HHs)**					
	Daily (2 or more times)	Daily (1 time)	3–6 d a week	1–2 d a week	Once or twice a month	Never	Daily (2 or more times)	Daily (1 time)	3–6 d a week	1–2 d a week	Once or twice a month	Never
**Cereals and millets**	**149 (93.1)**	1 (0.6)	8 (5.0)	2 (1.3)	—	—	**91 (91.0)**	3 (3.0)	5 (5.0)	1 (1.0)	—	—
*Indigenous cereals and millets*	39 (24.4)	1 (0.6)	9 (5.6)	12 (7.5)	14 (8.8)	**85 (53.1)**	**61 (61.0)**	5 (5.0)	7 (7.0)	—	3 (3.0)	24 (24.0)
**Pulses**	—	3 (1.9)	55 (34.3)	**101 (63.2)**	1 (0.6)	—	5 (5.0)	4 (4.0)	32 (32.0)	**47 (47.0)**	11 (11.0)	1 (1.0)
*Indigenous pulses*	—	1 (0.6)	13 (8.1)	**90 (56.3)**	11 (6.9)	45 (28.1)	3 (3.0)	1 (1.0)	24 (24.0)	**38 (38.0)**	14 (14.0)	20 (20.0)
**Green leafy vegetables**	1 (0.6)	1 (0.6)	60 (37.5)	**91 (56.9)**	6 (3.8)	1 (0.6)	6 (6.0)	4 (4.0)	40 (40.0)	**44 (44.0)**	4 (4.0)	2 (2.0)
*Indigenous green leafy vegetables*	1 (0.6)	1 (0.6)	53 (33.3)	**91 (56.9)**	7 (4.3)	7 (4.3)	2 (2.0)	4 (4.0)	11 (11.0)	**41 (41.0)**	22 (22.0)	20 (20.0)
**Other vegetables**	1 (0.6)	11 (6.9)	**80 (50.0)**	65 (40.6)	2 (1.3)	1 (0.6)	**38 (38.0)**	20 (20.0)	22 (22.0)	16 (16.0)	4 (4.0)	—
*Indigenous vegetables*	1 (0.6)	3 (1.9)	17 (10.6)	41 (25.6)	18 (11.2)	**80 (50.0)**	2 (2.0)	1 (1.0)	20 (20.0)	25 (25.0)	17 (17.0)	**35 (35.0)**
**Roots and tubers**	**123 (76.9)**	27 (16.9)	1 (0.6)	3 (1.9)	1 (0.6)	5 (3.1)	**88 (88.0)**	9 (9.0)	1 (1.0)	2 (2.0)	—	—
*Indigenous roots and tubers*	—	—	4 (2.5)	31 (19.4)	22 (13.7)	**103 (64.4)**	—	1 (1.0)	7 (7.0)	10 (10.0)	35 (35.0)	**47 (47.0)**
**Fruits**	38 (23.7)	**46 (28.7)**	37 (23.1)	34 (21.3)	2 (1.3)	3 (1.9)	3 (3.0)	2 (2.0)	12 (12.0)	28 (28.0)	**35 (35.0)**	20 (20.0)
*Indigenous fruits*	1 (0.6)	2 (1.3)	3 (1.9)	19 (11.8)	25 (15.6)	**110 (68.8)**	3 (3.0)	2 (2.0)	6 (6.0)	10 (10.0)	18 (18.0)	**61 (61.0)**
**Milk and milk products** ^2^	—	8 (5.0)	3 (1.9)	4 (2.5)	4 (2.5)	**141 (88.1)**	4 (4.0)	1 (1.0)	3 (3.0)	—	2 (2.0)	**90 (90.0)**
**Meat, fish, and poultry**	2 (1.3)	2 (1.2)	15 (9.4)	**87 (54.3)**	50 (31.3)	4 (2.5)	1 (1.0)	—	6 (6.0)	**57 (57.0)**	35 (35.0)	1 (1.0)
*Indigenous meat, fish, and poultry*	2 (1.3)	1 (0.6)	1 (0.6)	15 (9.4)	**85 (53.1)**	56 (35)	—	—	2 (2.0)	14 (14.0)	39 (39.0)	**45 (45.0)**
**Mushrooms (indigenous)**	—	1 (0.6)	34 (21.2)	**101 (63.2)**	15 (9.4)	9 (5.6)	—	—	—	—	—	—
**Oils and fats**	**155 (96.9)**	1 (0.6)	4 (2.5)	—	—	—	**91 (91.0)**	4 (4.0)	1 (1.0)	3 (3.0)	1 (1.0)	—
*Indigenous oils*	5 (3.1)	—	1 (0.6)	4 (2.5)	—	150 (93.8)	**9 (9.0)**	—	1 (1.0)	1 (1.0)	—	89 (89.0)
**Sugar** ^2^	5 (3.1)	23 (14.4)	43 (26.9)	**70 (43.8)**	12 (7.5)	7 (4.3)	13 (13.0)	17 (17.0)	17 (17.0)	**28 (28.0)**	9 (9.0)	16 (16.0)
**Market procured packaged and fresh foods** ^2^	6 (3.8)	31 (19.4)	**98 (61.2)**	25 (15.6)	—	—	17 (17.0)	22 (22.0)	**34 (34.0)**	27 (27.0)	—	—

Note: Figures in bold indicate most frequent consumption.

HH, household.

1 Some frequency categories have been merged for easy readability.

2 No indigenous food items reported in the food group.

### Anthropometric assessment of Munda women

**FIGURE 3:**
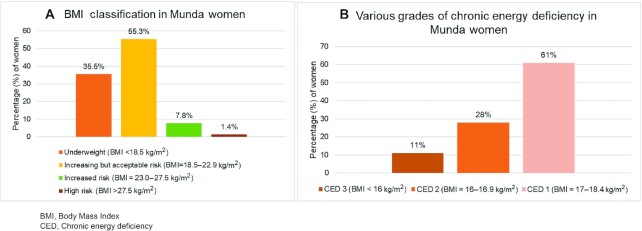
A. BMI classification (*n* = 282); B. Various grades of CED classification (*n*  = 100) in Munda women of Jharkhand, India.

The nutritional status of the women (*n* = 282) based on anthropometric assessments, showed that more than one-third of the women (35%) had CED, and among these women, 11% were found to be in the category of CED III (<16 kg/m^2^) (**Figure 3**). There was no association found between BMI and usual nutrient intake of Munda women (*P*> 0.05).

### Nutrient intake of Munda women (18–49 y)

**Table 3** describes the mean usual nutrient intakes of the Munda women across seasons as well as the proportion of women with usual intakes below the recommendations (EAR). During the monsoon season (*n* = 282), the usual nutrient intakes of all women were less than the Indian EAR values for all nutrients except for protein. This was consistent in the winter season (*n* = 118) as well, with usual nutrient intakes lower than EAR in almost all women for all nutrients, except for protein and vitamin C. Although low intakes were observed in both seasons, the usual nutrient intakes for all the macro- and micronutrients were significantly higher (*P* < 0.001) in winter compared with monsoon season.

**TABLE 3:** Mean daily usual nutrient intakes of women (18–49 y) and prevalence of nutrient inadequacy across seasons, in Munda tribal community of Jharkhand, India

**Nutrients**	**EAR**^1^	**Wet monsoon (*n* = 282)**	**Winter (*n* = 118)**		**% population with inadequate intake**	
					**Wet monsoon (*n* = 282)**	**Winter (*n* = 118)**
		Mean ± SD	Mean ± SD	*P* value^2^	*n* < EAR (%)	*n* < EAR (%)
Energy, kcal/d	2130	1305 ± 195.1	1614 ± 343.5	**<0.001**	282 (100.0)	108 (91.5)
Protein, g/d	36	33.5 ± 3.9	37.8 ± 6.7	**<0.001**	215 (76.2)	44 (37.2)
Fat, g/d	NA	9.3 ± 2.2	13.1 ± 4.8	**<0.001**	NA	NA
Carbohydrate, g/d	NA	239.9 ± 33.3	315.9 ± 67.1	**<0.001**	NA	NA
Total dietary fiber, g/d	NA	17.04 ± 2.05	22.8 ± 6.13	**<0.001**	NA	NA
Vitamin A, µg/d	390	22.1 ± 12.6	115.4 ± 56.8	**<0.001**	282 (100.0)	118 (100.0)
Thiamine, mg/d	1.4	0.4 ± 0.1	0.5 ± 0.1	**<0.001**	282 (100.0)	118 (100.0)
Riboflavin, mg/d	2.0	0.3 ± 0.1	0.4 ± 0.2	**<0.001**	282 (100.0)	118 (100.0)
Niacin, mg/d	12.0	6.6 ± 0.8	7.8 ± 2.2	**<0.001**	282 (100.0)	114 (96.6)
Pyridoxine, mg/d	1.6	0.7 ± 0.1	0.8 ± 0.2	**<0.001**	282 (100.0)	118 (100.0)
Folate, µg/d	180	89.9 ± 8.5	138.4 ± 19.2	**<0.001**	282 (100.0)	116 (98.3)
Vitamin C, mg/d	55	29.9 ± 3.9	68 ± 14.1	**<0.001**	282 (100.0)	21 (17.8)
Iron, mg/d	15.0	5.8 ± 1	8.2 ± 2.3	**<0.001**	NA^3^	(100.0)^3^
Calcium, mg/d	800.0	95.1 ± 9.6	146.4 ± 5.6	**<0.001**	282 (100.0)	118 (100.0)
Zinc, mg/d	11.0	5.2 ± 0.8	5.9 ± 1.2	**<0.001**	282 (100.0)	117 (99.2)

Note: Bold numbers indicate significance at *P* value < 0.001.

EAR, estimated average requirements; NA, not available.

1 EAR value available in the Recommended Dietary Allowances for Indians (51).

2 Paired *t*-test was used to determine the significance of variation in mean daily nutrient intake in the 2 seasons.

3 % nutrient inadequacy for iron was calculated using the probability approach (92).

### Seasonal dietary diversity and its contribution to nutrient intake in Munda women

#### Wet monsoon season

**FIGURE 4:**
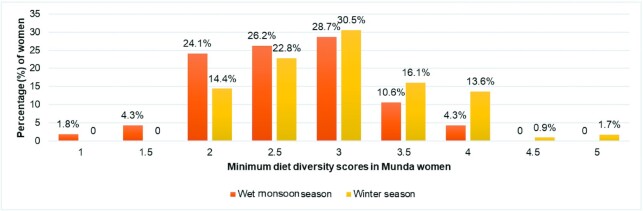
Distribution of MDDS across two seasons in Munda women of Jharkhand, India.

The mean MDDS of women during monsoon season (*n* = 282) was found to be 2.6 ± 0.6 (minimum—1, maximum—4), with 54% women having an MDDS between 2.5 and 3 (**Figure 4**). The consumption of a minimum diverse diet (i.e. MDDS ≥5) was not observed in any women. Nonetheless, even at these low levels of dietary diversity, women having a higher MDDS were found to have significantly higher (*P* < 0.001) usual intakes of all nutrients studied (**Table 4**). Further, MDDS was not significantly different across all quintiles of the HH Wealth Score Index except between the lowest and upper middle quintile (*P* < 0.05). In addition, MDDS did not differ across the education level of the Munda women (*P* > 0.05).

**TABLE 4:** Correlation of IF consumption and MDDS with usual nutrient intakes in wet monsoon (*n* = 282) and winter season (*n* = 118), Munda tribal women, India

**Nutrients**	**IF consumption scores, *r*^1^**		**MDDS, *r*^1^**	
	Wet monsoon season	Winter season	Wet monsoon season	Winter season
Energy	0.07	0.05	0.30***	0.23*
Protein	0.02	0.14	0.40***	0.3***
Fat	−0.03	−0.07	0.43***	0.33***
Carbohydrate	0.08	0.09	0.18**	**0.17**
Dietary fiber	0.09	0.06	0.34***	0.35***
Vitamin A	0.29***	0.17	0.20***	0.14
Thiamine	0.02	0.34***	0.36***	0.28**
Riboflavin	0.20***	0.32***	0.45***	0.11
Niacin	0.05	0.13	0.33***	0.02
Pyridoxine	0.13*	0.12	0.27***	0.19*
Folate	−0.02	0.07	0.32***	0.41***
Vitamin C	0.13*	−0.02	0.25***	−0.01
Iron	0.06	−0.02	0.39***	0.37***
Calcium	0.18**	−0.03	0.28***	0.29**
Zinc	0.05	0.19*	0.36***	0.19*

Note: Bold numbers indicate significance.

IF, Indigenous foods.

MDDS, Minimum Dietary Diversity Score for women.

1 Karl Pearson's correlation coefficient was used to determine the significance of the association of IF consumption and MDDS with usual nutrient intakes.

*P < 0.05, **P < 0.01, ***P < 0.001.

#### Winter season

During winters, compared with the monsoon season, significantly higher dietary diversity scores (*P* < 0.001) were reported, with a mean MDDS of 3 ± 0.7 (minimum—2, maximum—5). Although a few women (14%) reported the consumption of 4 food groups during the 2-d 24-h DR period, only 2 out of 118 women surveyed in the season consumed a minimally diverse diet (MDDS ≥5). Higher MDDS during winters were also seen to be positively associated with a higher usual intake of energy (*P* < 0.05), protein (*P* < 0.001), fat (*P* < 0.001), dietary fiber (*P* < 0.001), thiamine (*P* < 0.01), pyridoxine (*P* < 0.05), folate (*P* < 0.001), iron (*P* < 0.001), calcium (*P* < 0.01), and zinc (*P* < 0.05) (Table 4).

### Indigenous food consumption and its contribution to usual nutrient intake in Munda women

#### Wet monsoon season

**FIGURE 5:**
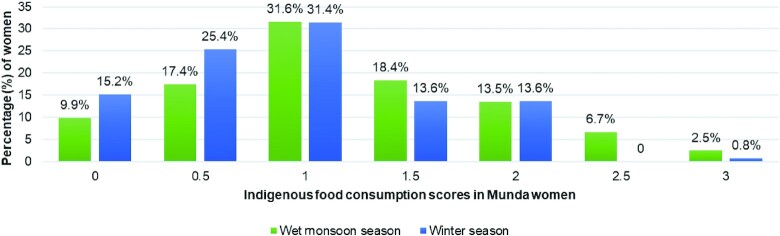
Distribution of IF consumption scores across two seasons in Munda women of Jharkhand, India.

About 73% of women in the monsoon season reported IF consumption during the 2-d 24 -h DR period, with a mean IF consumption score of 1.2 ± 0.7. Among these, about half the women (50%) had IF scores ranging between 1 and 1.5 (**Figure 5**). Upon further exploration of the types and amounts of IFs consumed by the women, Indigenous vegetables (including mushrooms) were found to be the most reported food item during the monsoon season, although the median intake consumed per day was found to be quite low [5.2% of the recommended dietary intakes (RDIs) for moderately active Indian women] (51) (**Table 5**). The consumption of Indigenous rice varieties was reported by 40% women, with a relatively higher median intake per day (58.8% of RDI). Lower intakes were reported for Indigenous fruits (5.8% of RDI), pulses (18.6% of RDI), roots and tubers (20.8% of RDI), GLVs (27.8% of RDI), and flesh foods (26.7% of RDI), which were consumed by very few women. Despite overall poor IF intakes, women with higher IF scores were found to have significantly higher usual intakes of vitamin A (*P* < 0.001), riboflavin (*P* < 0.001), pyridoxine (*P* < 0.05), vitamin C (*P* < 0.05), and calcium (*P* < 0.01) in the monsoon season (Table 4). IF consumption did not differ across the education level and quintiles of HH Wealth Score Index (*P* > 0.05).

**TABLE 5:** Food groupwise intakes reported in the 2-d 24-h dietary recall, among adult Munda women, Jharkhand, India

		Wet monsoon season (*n* = 282)			Winter season (*n* = 118)		
Food groups	Recommended Dietary Intake^1^ (g/d)	Median intake (IQR) (g/d)	Median intake of Indigenous foods (IQR) (g/d)	No. of women consuming the Indigenous food group *n* (%)	Median intake (IQR) (g/d)	Median intake of Indigenous foods (IQR) (g/d)	No. of women consuming the Indigenous food group *n* (%)
Cereals	300	288.4 (218.3–375)	176.5 (108.5–270.2)	115 (40.8)	338.5 (266.5–416.6)	138.7 (93.7– 245.3)	58 (49.2)
Pulses	90	22.9 (12.4–41.7)	16.7 (11.3–32.0)	67 (23.8)	31.3 (20.7–61.8)	32 (20.9–71.2)	46 (38.9)
Green leafy vegetables	150	41.7 (25.8–63.8)	41.6 (20.8–57.8)	123 (43.6)	62.5 (35.6–106.1)	15.7 (1.1–30.6)	42 (35.6)
Other vegetables (including mushrooms)	200	52.1 (26.1–93.8)	10.4 (0–43.6)	161 (56.9)	75.2 (41–126.4)	15.6 (10.2–26.4)	11 (9.3)
Roots and tubers	100	56.6 (31.2–98.3)	20.8 (15.6–23.9)	6 (2.1)	110.1 (64.5–189.6)	55.5 (43.9–86.7)	7 (5.9)
Fruits	150	8.7 (3.1–23.3)	8.7 (8.7–8.7)	1 (0.4)	33.3 (6.2–69)	33.3 (22.5–61.1)	3 (2.5)
Flesh foods (meat, fish, and poultry)	90	37.5 (22.5–72.8)	24.0 (14.1–33.0)	11 (3.9)	40 (27.5–86)	32.0 (12.8–52.3)	4 (3.3)

1 Recommended Dietary Intake values for moderately active Indian woman (51).

#### Winter season

During winters, compared with the monsoon season, significantly lower (*P* < 0.001) IF scores were reported, with a mean score of 0.9 ± 0.6. About 59% women reported IF consumption, with 31.4% of them having an IF score of 1 (Figure 5). Quantitative estimates of IF intake from different food groups were observed to be low in the winter season as well, especially for Indigenous GLVs (10.5% of RDI) and other vegetables (7.8% of RDI). However, in comparison to the monsoon season, a slightly higher proportion of Munda women reported the consumption of Indigenous rice (49.2%), pulses (38.9%), fruits (2.5%), and roots and tubers (5.9%), with a relatively better median intake per day for Indigenous pulses (35.6% of RDI), fruits (22.8% of RDI), and roots and tubers (55.5% of RDI) (Table 5). During winters, women with higher IF scores were found to have significantly higher usual intakes of thiamine (*P* < 0.001), riboflavin (*P* < 0.001), and zinc (*P* < 0.05) (Table 4).

### Association of Indigenous food consumption with usual nutrient intake of Munda women

In the present study, dietary diversity, educational profile of women, and HH Wealth Score Index were taken as confounding variables to test the association between IF consumption and usual nutrient intakes of Munda women. The adjusted findings obtained after considering the effect of confounding variables, are presented in **Table 6**. Estimates are presented in terms of unstandardized coefficients along with 95% CI in the independent variable. Multicollinearity was assessed using the variance inflation factor which was found to be <5. After controlling for other covariates in the model, IF consumption was found to be significantly associated with a higher usual intake of vitamin A (*P* < 0.001) and a lower usual intake of fat (*P* < 0.05) and folate (*P* < 0.05), respectively. On the other hand, dietary diversity (MDDS) was found to have a positive significant (*P* < 0.001) association with usual intakes of all nutrients studied. Moreover, women belonging to HHs with better socieconomic status (upper middle and upper most quintiles) were seen to have a significantly (at *P* values < 0.05 and < 0.01 respectively) higher usual intake of fat, dietary fiber, folate, iron, and calcium, after controlling for other confounding variables (Table 6).

**TABLE 6:** Factors associated with usual intake among Munda women, Jharkhand, India (*n* = 282)

Characteristics		Energy	Protein	Fat	Carbohydrate	Dietary fiber	Vitamin A	Thiamine	Riboflavin	Niacin	Pyridoxine	Folate	Vitamin C	Iron	Calcium	Zinc
**IF consumption scores**	Unstandardized coefficients	−3.86	−0.44	−0.52***	**0.59**	**0.033**	2.58***	−0.0084	0.0034	−0.070	−0.0019	−1.27**	0.26	−0.073	0.47	−0.069
	95% CI (lower, upper)	(–30.8, 23.1)	(–0.9, 0.02)	(–0.8, –0.2)	(–3.8, 5.01)	(–0.2, 0.3)	(1.4, 3.7)	(–0.02, 0.0)	(–0.0, 0.01)	(–0.2, 0.03)	(–0.01, 0.0)	(–2.2, –0.3)	(–0.4, 0.9)	(–0.2, 0.1)	(–0.7, 1.6)	(–0.2, 0.03)
**MDDS**	Unstandardized coefficients	74.1***	2.12***	1.46***	7.28**	0.81***	3.34***	0.036***	0.030***	0.38***	0.021***	3.88***	1.49***	0.55***	4.66***	0.37***
	95% CI (lower, upper)	(42.5, 105.7)	(1.6, 2.7)	(1.1, 1.8)	(2.1, 12.5)	(0.5, 1.1)	(1.9, 4.7)	(0.02, 0.1)	(0.02, 0.04)	(0.3, 0.5)	(0.02, 0.03)	(2.8, 4.9)	(0.7, 2.2)	(0.4, 0.7)	(3.3, 6.03)	(0.3, 0.5)
**HH Wealth Score Index** ^1^																
Lowest middlequintile	Unstandardized coefficients	28.0	0.29	0.16	2.85	0.28	−1.56	0.0059	−0.0073	0.18	0.0053	0.84	−0.64	0.13	1.51	0.089
	95% CI (lower, upper)	(–31.3, 87.4)	(–0.7, 1.3)	(–0.5, 0.8)	(–6.9, 12.6)	(–0.3, 0.9)	(–4.1, 0.9)	(–0.02, 0.03)	(–0.02, 0.01)	(–0.05, 0.41)	(–0.01, 0.02)	(–1.2, 2.9)	(–1.9, 0.7)	(–0.2, 0.4)	(–1.1, 4.1)	(–0.1, 0.3)
Lower middlequintile	Unstandardized coefficients	9.33	−0.069	−0.0093	−2.97	0.32	−1.63	0.0019	−0.00047	−0.023	−0.0016	0.74	−1.30	0.31	1.02	0.077
	95% CI (lower, upper)	(–52.8, 71.5)	(–1.1, 1.0)	(–0.7, 0.7)	(–13.16, 7.23)	(–0.28, 0.93)	(–4.3, 1.04)	(–0.0, 0.04)	(–0.02, 0.01)	(–0.3, 0.2)	(–0.01, 0.01)	(–1.4, 2.9)	(–2.7, 0.1)	(–0.01, 0.6)	(–1.7, 3.7)	(–0.1, 0.3)
Upper middlequintile	Unstandardized coefficients	32.8	0.60	0.72*	−0.44	0.66*	−0.88	0.0017	−0.00020	0.042	0.0022	2.38*	−1.21	0.37*	2.26	0.15
	95% CI (lower, upper)	(–31.02, 96.6)	(–0.5, 1.7)	(0.0, 1.4)	(–10.9, 10.04)	(0.04, 1.3)	(–3.6, 1.8)	(–0.01, 0.04)	(–0.02, 0.01)	(–0.2, 0.3)	(–0.01, 0.01)	(0.2, 4.6)	(–2.6, 0.2)	(0.04, 0.7)	(–0.5, 5.03)	(–0.1, 0.4)
Upper mostquintile	Unstandardized coefficients	33.3	0.52	1.10**	0.64	0.49	−0.82	0.0020	−0.0020	0.16	0.0031	3.28**	−0.83	0.42*	3.47*	0.16
	95% CI (lower, upper)	(–33.02, 99.6)	(–0.6, 1.7)	(0.4, 1.8)	(–10.3, 11.5)	(–0.2, 1.1)	(–3.7, 2.04)	(–0.0, 0.04)	(–0.02, 0.01)	(–0.1, 0.4)	(–0.01, 0.02)	(0.9, 5.6)	(–2.3, 0.6)	(0.1, 0.8)	(0.6, 6.3)	(–0.1, 0.4)
**Education level** ^2^																
Above primary level but below secondary	Unstandardized coefficients	11.7	0.078	0.37	0.88	0.22	−0.63	0.0048	0.0010	0.015	0.0011	0.12	0.54	−0.041	−0.25	0.0084
	95% CI (lower, upper)	(–34.5, 57.8)	(–0.7, 0.9)	(–0.16, 0.9)	(–6.7, 8.4)	(–0.23, 0.67)	(–2.6, 1.4)	(–0.01, 0.02)	(–0.01, 0.01)	(–0.2, 0.2)	(–0.01, 0.01)	(–1.5, 1.7)	(–0.5, 1.6)	(–0.3, 0.2)	(–2.3, 1.7)	(–0.2, 0.2)
Secondary and above	Unstandardized coefficients	10.7	0.72	0.65*	−5.56	0.47	0.30	0.025*	0.011	0.035	0.0054	0.32	0.71	0.23	−0.59	0.11
	95% CI (lower, upper)	(-43.9, 65.3)	(–0.2, 1.7)	(0.02, 1.3)	(–14.5, 3.4)	(–0.1, 1.0)	(–2.1, 2.6)	(0.01, 0.04)	(–0.0, 0.02)	(–0.2, 0.3)	(–0.01, 0.02)	(–1.6, 2.2)	(–0.6, 1.9)	(–0.1, 0.5)	(–2.9, 1.8)	(–0.1, 0.3)

Note: Bold numbers indicate significance.

IF, Indigenous foods.

MDDS, Minimum Dietary Diversity Score.

HH, Household.

1 HH Wealth Score Index lowest quintile.

2 Education level primary and below.

*P < 0.05, **P < 0.01, ***P < 0.001.

### Seasonal variation in Indigenous food consumption and usual nutrient intake of Munda women

In order to determine the impact of seasonal variation on usual intake of study participants, a linear mixed effect regression model was employed using season as a fixed factor along with other covariates mentioned above; the results are presented in **Table 7**. Overall, season was found to be a statistically significant predictor of the model. We found a significant association of IF consumption with a higher usual intake of riboflavin (*P* < 0.001) and lower intake of fat (*P* < 0.05), respectively. Dietary diversity (MDDS) was also found to have a significant (*P* < 0.001) association with higher usual intakes of all nutrients, except carbohydrate, vitamin C, riboflavin, and niacin. Additionally, women with the highest level of education were found to have a significantly (*P* < 0.05) higher usual intake of macro- (protein, fat, and dietary fiber) as well as micronutrients (folate, pyridoxine, thiamine, riboflavin, and zinc).

**TABLE 7:** Factors associated with usual intake after adjusting for seasonality among Munda women, Jharkhand, India (*n* = 118)

Characteristics		Energy	Protein	Fat	Carbohydrate	Dietary fiber	Vitamin A	Thiamine	Riboflavin	Niacin	Pyridoxine	Folate	Vitamin C	Iron	Calcium	Zinc
**IF consumption scores**	Unstandardized coefficients	5.88	0.30	−0.76*	5.38	0.049	2.20	0.013	0.032***	0.16	0.0064	−0.96	−0.73	−0.21	0.094	0.11
	95% CI (lower, upper)	(–44.7, 56.5)	(–0.7, 1.2)	(–1.4, –0.1)	(–4.7, 15.5)	(–0.7, 0.8)	(–4.9, 9.3)	(–0.0, 0.03)	(0.01, 0.1)	(–0.2, 0.5)	(–0.01, 0.03)	(–3.5, 1.6)	(–2.6, 1.2)	(–0.5, 0.1)	(–1.3, 1.5)	(–0.1, 0.3)
**MDDS**	Unstandardized coefficients	78.5**	2.22***	1.78***	9.81	1.36**	13.7***	0.028**	0.017	0.30	0.038**	6.93***	1.55	0.60***	3.19***	0.29**
	95% CI (lower, upper)	(23.5, 133.4)	(1.2, 3.3)	(1.1, 2.5)	(–1.3, 20.9)	(0.5, 2.2)	(5.9, 21.5)	(0.01, 0.1)	(–0.0, 0.04)	(–0.1, 0.7)	(0.02, 0.1)	(4.1, 9.8)	(–0.6, 3.7)	(0.3, 0.9)	(1.6, 4.8)	(0.1, 0.5)
**Seasonality**	Unstandardized coefficients	270.9***	3.36***	2.71***	72.4***	5.24***	88.5***	0.099***	0.075***	1.16***	0.10***	45.1***	37.5***	2.14***	50.3***	0.60***
	95% CI (lower, upper)	(209.8, 332.04)	(2.2, 4.6)	(1.9, 3.6)	(59.6, 85.3)	(4.2, 6.3)	(78.9, 98.1)	(0.1, 0.1)	(0.05, 0.10)	(0.7, 1.6)	(0.1, 0.1)	(41.6, 48.5)	(34.9, 40.1)	(1.8, 2.5)	(48.3, 52.3)	(0.4, 0.9)
**HH Wealth Score Index** ^1^																
Lowest middlequintile	Unstandardized coefficients	35.3	0.63	0.012	11.6	0.24	−9.80	0.018	−0.0095	−0.20	−0.011	0.33	−0.26	0.020	−0.61	0.15
	95% CI (lower, upper)	(–90.5, 161.02)	(–1.6, 2.8)	(–1.5, 1.5)	(–12.1, 35.3)	(–1.4, 1.9)	(–25.4, 5.8)	(–0.02, 0.1)	(–0.1, 0.03)	(–0.9, 0.5)	(–0.1, 0.03)	(–5.3, 5.9)	(–4.5, 3.9)	(–0.6, 0.6)	(–3.7, 2.5)	(–0.3, 0.6)
Lower middlequintile	Unstandardized coefficients	−43.4	−0.52	−0.65	−7.63	−0.095	−20.3*	0.036	0.0083	−0.31	−0.012	0.097	−1.13	0.063	1.02	0.051
	95% CI (lower, upper)	(–168.6, 81.9)	(–2.7, 1.7)	(–2.2, 0.8)	(–31.3, 16.02)	(–1.8, 1.6)	(–35.8, –4.9)	(–0.0, 0.1)	(–0.03, 0.1)	(–1.01, 0.4)	(–0.1, 0.03)	(–5.5, 5.7)	(–5.3, 3.0)	(–0.5, 0.6)	(–2.1, 4.1)	(–0.4, 0.5)
Upper middlequintile	Unstandardized coefficients	−3.26	0.29	0.21	−3.88	0.20	−19.9*	0.028	−0.00052	−0.39	−0.010	1.98	−0.13	0.21	1.48	0.17
	95% CI (lower, upper)	(–145.9, 139.4)	(–2.2, 2.8)	(–1.5, 1.9)	(–30.3, 22.6)	(–1.7, 2.1)	(–37.8, –2.1)	(–0.01, 0.1)	(–0.04, 0.04)	(–1.2, 0.4)	[–0.1, 0.04)	(–4.4, 8.3)	(–4.9, 4.6)	(–0.5, 0.9)	(–1.8, 4.7)	(–0.3, 0.6)
Upper mostquintile	Unstandardized coefficients	−72.8	−0.99	−0.29	−11.9	−1.14	−20.1*	0.023	−0.012	−0.64	−0.042	−2.44	−0.95	−0.13	0.97	−0.021
	95% CI (lower, upper)	(–207.03, 61.4)	(–3.4, 1.4)	(–1.89, 1.3)	(–36.9, 13.1)	(–2.9, 0.1)	(–36.8, –3.3)	(–0.02, 0.1)	(–0.05, 0.03)	(–1.4, 0.1)	(–0.1,0.0)	(–8.4, 3.5)	(–5.4, 3.5)	(–0.8, 0.5)	(–2.2, 4.1)	(–0.5, 0.4)
**Education level** ^2^																
Above primary level but below secondary	Unstandardized coefficients	33.6	−0.42	1.01	2.2	0.59	6.03	0.011	0.0083	0.074	0.013	1.48	3.08	0.040	−1.76	−0.042
	95% CI (lower, upper)	(–60.2, 127.4)	(–2.1, 1.2)	(–0.13, 2.1)	(–15.6, 19.9)	(–0.7, 1.9)	(–5.7, 17.8)	(–0.02, 0.04)	(–0.02, 0.04)	(–0.5, 0.6)	(–0.02, 0.1)	(–2.8, 5.7)	(–0.1, 6.2)	(–0.4, 0.5)	(–4.1, 0.6)	(–0.4, 0.3)
Secondary and above	Unstandardized coefficients	98.5	2.61**	1.40*	8.91	1.52*	−1.30	0.040*	0.046*	0.51	0.042*	6.23*	1.33	0.47	0.13	0.43*
	95% CI (lower, upper)	(–12.7, 209.7)	(0.7, 4.6)	(0.1, 2 .7)	(–12.1, 29.9)	(0.03, 3.01)	(–15.1, 12.5)	(0.01, 0.1)	(0.01, 0.1)	(–0.1, 1.1)	(0.0, 0.1)	(1.3, 11.2)	(–2.4, 5.03)	(–0.04, 0.9)	(–2.6, 2.9)	(0.1, 0.8)

Note: Bold numbers indicate significance.

IF, Indigenous foods.

MDDS, Minimum Dietary Diversity Score.

HH, Household.

1 HH lowest quintile.

2 Education level primary and below.

*P < 0.05, **P < 0.01, ***P < 0.001.

## Discussion

In this study, we have explored the dietary intake and nutritional status of Munda tribal women of Jharkhand in the context of their IF environment, dietary diversity, and socioeconomic and demographic profiles. The community demonstrated a poor socioeconomic and demographic profile with limited diversity in the types of foods accessed from the natural food environment. The poor access to diverse foods was reflected in poor dietary and nutrient intakes of the tribal women during both seasons and a high prevalence of CED (BMI <18.5 in 35% women). Both the IF consumption and MDDS were low in both seasons; however, even at low scores, IF consumption and MDDS were positively associated with a higher usual intake of macro- and micronutrients among Munda women.

### Availability of a diverse natural food environment but poor household food access

The Munda tribal community was aware of the availability of diverse natural food sources, a feature commonly observed across tribal communities of Jharkhand, a state endowed with rich biodiversity including diverse physiographic, agroecological habitats, geographical regions, and climatic conditions (23, 57–60). However, it was intriguing to see that that availability of biodiverse food sources was not translated into actual food access. Our quantitative estimates on HH access to foods derived from the natural food environment (FADI score) in this community was not very encouraging. The disparity in biodiverse food sources versus actual HH access has also been demonstrated in other tribal communities in India and elsewhere (11, 23, 58–62). The possible reasons attributed for this disparity include rapid nutrition transition marked with better market access, the high opportunity cost of accessing natural food sources, forest conservation laws, and gradual erosion of transgenerational TEK (6, 58, 60, 63).

The Munda community is comprised mostly of settled smallholder farmers who reported growing mostly monocrops (rice), along with some pulses such as red gram, black gram, and horse gram. Only a handful of these farmers were growing Indigenous varieties of crops, predominantly, rice. Another study by our team has reported the historical use of Indigenous millets in this community but the practice has ceased, which was attributed to climate variability and resulting poor access to natural resources, especially water (23). Our previous studies in Jharkhand have also documented high nutritive values in Indigenous crops and foods that are consumed by tribal communities in the region, thus highlighting their potential for addressing malnutrition (23, 58–60). A study in Jharkhand has documented the collective wealth of Indigenous knowledge for agricultural practices and its components such as land preparation, sustainable management of water resources etc., among the local tribal communities. The study further highlighted the importance of capacity building of the smallholder farmers on participatory monitoring, conservation, and optimal utilization of the natural resources base while integrating Indigenous crop production systems with modern methods and technologies for resource conservation (64). Programs incorporating such initiatives have the potential to address malnutrition through nutrition-sensitive and sustainable agriculture.

### Household access to market and poor outreach of government food security and supplementation programs

The community reported good access to an informal built food environment (i.e. local markets) and suboptimal access to government food security and food supplementation programs. Almost a quarter of the HHs in the Munda community were not accessing the TPDS. This is in line with the findings of a latest report which has pointed toward 80% coverage of the TPDS in the state of Jharkhand (65). Studies documenting poor access and utilization of food commodities from TPDS by tribal communities in the states of Uttar Pradesh, Maharashtra, Kerala, and Andhra Pradesh highlighted reasons such as non-preference toward the standard cereals (wheat/rice) distributed in TPDS versus the preferred traditional coarse grains such as Ragi (finger millet), Bajra (pearl millet), and other millets, poor access to the ration shops among tribal communities residing in difficult terrains, and governance issues and pilferage in the supply chain (66–69). On the other hand, we have also learned from a state level program like the “Anna Bhagya” scheme in the state of Karnataka, which intended to give due importance to regional food security by providing food commodities under the TPDS according to geographic regions and local preferences for traditional grains (70).

### Poor household food access leading to poor dietary diversity in women

The poor access diversity with low mean FADI score translated into poor dietary diversity (MDDS) among Munda women. Low indices of dietary diversity in women have also been observed in the Sauria Paharia tribe of Jharkhand and Khasi tribe of Meghalaya (71). Although in our study, dietary diversity scores of the women were not significantly associated with their literacy profile and HH Wealth Index (except for the lowest and upper middle quintile), studies from other parts of India have identified significant associations between higher dietary diversity with higher family education, higher annual income, greater crop diversity, and increased distance travelled to markets (72, 73). Similarly, a study conducted in rural and urban parts of Uttar Pradesh found a significant and positive association of HH income, literacy, and occupation of the head of the HH with the HH dietary diversity, whereas family size had a negative influence (74). Factors like poor access to land and lack of nutrition knowledge could be additional possible reasons for poor dietary diversity among our study population (75, 76). In this context, good practices are documented from developing countries like Nepal where interventions to diversify food access such as the enhanced homestead food production program, has demonstrated its potential to address dietary diversity constraints among their rural population (77).

### Poor dietary diversity and limited Indigenous food consumption in women associated with poor nutritional intake

The low food access diversity and dietary diversity were further reflected in the poor intake of nutrient-dense IFs and usual nutrient intake of Munda women. The reported lower intake of key nutrients like energy, protein, vitamins A, B vitamins, C, iron, and zinc was comparable to the intakes reported in other tribal communities of Jharkhand and other states (10, 11, 13, 25, 39, 61, 62, 78–80). It was also observed that the risk of nutrient inadequacy in Munda women was 100% for all nutrients, except protein (in both seasons) and vitamin C (in winters). The lower risk of protein inadequacy could be attributed to a substantial amount of rice consumption which constituted a major portion of the main meals as well as the consumption of indigenous legumes (by 24–40% women in both seasons) on the previous day of recall; this may have contributed to a high aggregated protein intake. However, there is a likelihood that this adequate protein intake from a predominantly rice-based diet was perhaps of inferior quality, with suboptimal amino acid profile and low protein digestibility (81). On the other hand, the consumption of good quality protein like meat, poultry, fish, and milk products was almost negligible during both seasons: a pattern also observed in other tribal communities of India (10, 39, 62, 71, 78). Further, the lower risk of vitamin C inadequacy during the winter season could perhaps be due to a relatively higher intake of GLVs, other vegetables, and roots and tubers, which are usually rich sources of vitamin C (46). The usual intakes of both macro- and micronutrients were also seen to be significantly higher in the winter season, compared with the monsoon season, which could be due to the higher intake of all food groups during the winters; although the consumption amounts were grossly low in comparison to the RDI for moderately active Indian women (51). We also observed that the women were actively engaged in agricultural activities during the monsoon season, which may have compromised their cooking and food preparation activities at the HH level.

Although the Munda tribe displayed appreciable TEK of their IFs [in another study conducted by our group (23)], our findings revealed a possible discrepancy in the present knowledge and availability of IFs versus their actual intake at both the individual level and HH level. Studies have explored the potential reasons for this paradox in accessibility versus actual consumption of traditional IFs in tribal communities. The potential reasons cited include, high opportunity cost of accessing and preparation, lower value attributed to IFs (poor man's food), easy access, and preference for foods from markets and easy access to government food security programs (23, 58, 82, 83). However, it is worth noting that the IF consumption reported in Munda women (mostly as Indigenous varieties of rice, GLVs, mushrooms, other vegetables) even in limited quantities, contributed to a better intake of riboflavin even after adjusting for HH socioeconomic profile, women's literacy status, and seasonality. The high intake of specific micronutrients upon consumption of these IFs could be attributed to high nutrient density for iron, vitamin A, calcium, zinc, and folic acid in these foods, as documented in another article by our group (23). Globally, studies on Indigenous communities in Africa, Asia, and Latin America have also demonstrated better intake of protein, fiber, vitamin A, iron, calcium, and riboflavin in women with a higher IF intake (84–86). Apart from providing various nutritional benefits, IFs are also known to contain natural bioactive components with anti-inflammatory properties and may thus, have the potential to address the risk factors of several diet-related non-communicable diseases (another manifestation of malnutrition) (87, 88). Therefore, IFs could also be useful in addressing the growing concern around the overconsumption of narrowly diversified diets that provide empty calories in the absence of nutritional quality.

The women in the Munda community had poor nutritional status with 35% of them being underweight, a finding similar to previous studies on tribal women of Jharkhand and other states (10–12, 61, 62, 89, 90). The tribe-specific data generated in this study, thus, provides a detailed picture of the nutritional vulnerability of the community as well as the potential of utilizing traditional knowledge and the natural food environment as part of ongoing nutrition-specific and -sensitive programs.

Various organizations have implemented successful intervention projects in tribal areas of India to address the high burden of health and nutrition issues. In Odisha and Rajasthan, interventions have been aimed at reviving the use of rarely consumed wild foods among tribal communities for improved dietary diversity at the HH level (91). Another project in tribal regions of Odisha and Maharashtra improved HH diversity by increasing the availability and production of nutrient-dense crops (millets and pulses), promoting crop diversification, and supporting nutrition gardens of naturally biofortified fruits and vegetables, poultry farming, and fishery (91). In this context, the good practices documented for other tribal communities in India, could be applied to our study community, to diversify their food basket and improve their overall nutrient uptake.

### Study limitations

There are few study limitations that need to be highlighted. First, as the nutrient intakes were calculated using a software based on IFCT (46), which provides nutritive values of only raw Indian food items, the impact of cooking, food preservation, storage, and interactions between nutrients on their bioavailability were not accounted for, while assessing the nutrient intake of the study population. Second, vitamin B-12 intake could not be assessed in the study population, as the IFCT (46) provides no information on the vitamin B-12 content of raw Indian foods. Third, we expect some reporting and recall bias in the dietary assessment methods used (FFQ and 2 d 24-h DR). Fourth, despite using a food recall kit and a portion size estimation flip book, we expect some level of portion size estimation error in the recalls. Fifth, the FADI explored the foods accessed from the natural sources and did not consider the market foods as the purpose of this index was to explore the access to natural IF sources within these communities. Sixth, we administered a non-validated FFQ at the HH level, which may impact the generalizability of the study findings. Seventh, the MDDS calculation according to standard guidelines (44) was based on the intake of food items (≥15 g) and did not consider the nutrient density of the foods. Lastly, owing to the cross-sectional nature of the design, our study presents only associations, and no causal inference can be drawn from the findings. It is also possible that there could be unmeasured sources of confounding in this study.

## Conclusion

Despite living in a rich biodiverse food environment, there was poor access to diverse food sources and suboptimal consumption of balanced diets among Munda tribal women, thus contributing to high nutrient inadequacies. However, women who had better IF consumption and dietary diversity demonstrated better nutrient intakes, especially for micronutrients. There is scope to strengthen agricultural and food-based initiatives via the incorporation of IFs for production and consumption leading to effective, holistic, and sustainable solutions for tackling malnutrition among tribal women and the tribal communities in general, in India. These could involve restoring TEK about IF systems, promoting approaches for integrating traditional agricultural practices with new technologies to diversify food production, and enhancing the perceived value of IFs produced and collected using effective behavior change communication strategies for better consumption. At a programmatic level, food security programs could be strengthened by contextualizing their components to take into account traditional knowledge and local dietary practices and customizing interventions for diversifying the food baskets of Indigenous communities by the production, collection, and consumption of diverse foods utilizing the natural food environment. A better understanding of the local context in terms of diversity and cultural variation, understanding of Indigenous knowledge systems, and barriers and facilitators of IF consumption, can play a significant role in the effective implementation of nutrition programs. Although our study findings are specific for Munda women of Jharkhand and may not be generalizable, the factors that affected the food consumption and nutritional status in this community, could help in understanding the contribution of the IF environment in addressing malnutrition of other indigenous communities living in similar geographical terrains of India.

## Data Availability

Data described in the manuscript, code book, and analytic code will be made available upon request.

## Notes

This work was supported by the DBT (Department of Biotechnology)/Wellcome Trust India Alliance Fellowship (https://www.indiaalliance.org/) (IA/CPHI/16/1/502639) awarded to SG-J. The publication of this article was supported by the Shakopee Mdewakanton Sioux Community, through a gift to the University of Minnesota from its Seeds of Native Health campaign.

Author disclosures: The authors report no conflicts of interest.

Abbreviations used: CED, chronic energy deficiency; EAR, estimated average requirements; FADI, Food Accessed Diversity Index; GLV, green leafy vegetable; HH, household; IF, Indigenous food; IFCT, Indian Food Composition Table; MDDS, Minimum Diet Diversity Score for women; PCA, principal component analysis; PPS, probability proportional to size; RDI, recommended dietary intake; 24-h DR, 24-h dietary recall.
